# New Sign of Pregnancy

**Published:** 1846-10

**Authors:** 


14. *New Sign of Pregnancy.*—Dr. Pallender states that during a practice of 18 years, he has observed a peculiar smell of the vaginal mucus to be a constant and unerring sign of pregnancy. The smell is musty, something like that of spermatic fluid or liquor amnii; and, after a vaginal examination, it cannot be mistaken for any other odour. In a great many cases of pregnancy, during the first, second, and third months, when the condition of the patient was doubtful, owing to the earliness of the period, the author never, in a single instance, failed to discover the true state of the party by means of this sign. According to his latest observations, this odour is perceptible as early as the eighth day of gestation.—*American Journal*, in *Southern Med. and Surg. Journal.*

